# Mechanisms of Cellular Uptake of Cell-Penetrating Peptides

**DOI:** 10.1155/2011/414729

**Published:** 2011-04-07

**Authors:** Fatemeh Madani, Staffan Lindberg, Ülo Langel, Shiroh Futaki, Astrid Gräslund

**Affiliations:** ^1^Department of Biochemistry and Biophysics, Arrhenius Laboratories for Natural Sciences, Stockholm University, 10691 Stockholm, Sweden; ^2^Department of Neurochemistry, Arrhenius Laboratories for Natural Sciences, Stockholm University, 10691 Stockholm, Sweden; ^3^Institute for Chemical Research, Kyoto University, Uji, Kyoto 611-0011, Japan

## Abstract

Recently, much attention has been given to the problem of drug delivery through the cell-membrane in order to treat and manage several diseases. The discovery of cell penetrating peptides (CPPs) represents a major breakthrough for the transport of large-cargo molecules that may be useful in clinical applications. CPPs are rich in basic amino acids such as arginine and lysine and are able to translocate over membranes and gain access to the cell interior. They can deliver large-cargo molecules, such as oligonucleotides, into cells. Endocytosis and direct penetration have been suggested as the two major uptake mechanisms, a subject still under debate. Unresolved questions include the detailed molecular uptake mechanism(s), reasons for cell toxicity, and the delivery efficiency of CPPs for different cargoes. Here, we give a review focused on uptake mechanisms used by CPPs for membrane translocation and certain experimental factors that affect the mechanism(s).

## 1. Introduction

The cell membrane is the structure that protects living cells from the surrounding environment, only allowing the movement of compounds generally with small molecular size across this barrier into the cell. Some drugs are large hydrophilic molecules showing major limitations for their penetration through the cell membrane. A group of short peptides have been discovered that serve as delivery vectors for large molecules. They may have been called by different names such as protein translocation domain, membrane translocating sequence, Trojan peptide, or most commonly, cell-penetrating peptide (CPP).

Generally, CPPs are defined as short, water-soluble and partly hydrophobic, and/or polybasic peptides (at most 30–35 amino acids residues) with a net positive charge at physiological pH [1]. The main feature of CPPs is that they are able to penetrate the cell membrane at low micromolar concentrations *in vivo* and *in vitro* without using any chiral receptors and without causing significant membrane damage. Furthermore, and even more importantly, these peptides are capable of internalizing electrostatically or covalently bound biologically active cargoes such as drugs with high efficiency and low toxicity [1, 2]. 

This new class of peptides was introduced in the late 1980s by the discovery of the human immunodeficiency virus type 1 (HIV-1) encoded TAT peptide [3, 4] and the amphiphilic Drosophila *Antennapedia* homeodomain-derived 16 amino acid penetratin peptide (pAntp), which was discovered a few years later [5–8]. These two peptides are the most extensively studied of all CPPs. The mechanism(s) by which CPPs enter the cells has not been completely understood.

There is evidence for both energy-independent processes and endocytosis in internalization of CPPs. Presently, endocytosis, composed of two steps, endocytotic entry followed by endosomal escape, is believed to be the most common uptake mechanism at low CPP concentrations [2, 9].

## 2. Categories of CPPs

CPPs are categorized into the different subgroups based on their individual properties. One of the classifications is based on the origin of the peptide. It includes protein-derived peptides such as TAT and penetratin, which are also called protein transduction domains (PTDs). The second subgroup is the chimeric peptides which may contain two or more motifs from other peptides, for instance, transportan derived from mastoparan and galanin and its shorter analogue TP10. Synthetic peptides are another group in this category such as the polyarginine family [2, 16]. 

CPPs can also be divided into three other classes based upon different peptide sequences and binding properties to the lipids. These classes include primary amphipathic, secondary amphipathic and nonamphipathic CPPs [17]. Primary amphipathic CPPs (paCPPs), such as transportan [18] or TP10 [15] contain typically more than 20 amino acids. They have sequentially hydrophobic and hydrophilic residues along their primary structure [17]. In addition to endocytosis, the proposed mechanism for this group of CPPs is direct membrane transduction. Model studies have suggested that the direct transduction occurs via pore formation, carpet-like perturbations, or inverted micelles formed in the bilayer membrane. These mechanisms are described in [19]. Some primary amphiphatic CPPs such as TP10 are toxic to cells even at low concentrations. In addition, amphiphatic CPPs interact with both natural and anionic lipid membranes [17].

Secondary amphipathic CPPs (saCPPs), such as penetratin [7], pVEC [13], and M918 [14] often contain a smaller number of amino acids compared with primary amphiphatic CPPs. Their amphiphatic property is revealed when they form an alpha-helix or a beta sheet structure upon interaction with a phospholipid membrane. They typically bind to model membranes with a certain fraction of anionic lipids [17]. 

The third class, that is, the nonamphipathic peptides (naCPPs) are rather short with a high content of cationic amino acids (arginine) such as R9 [11] and TAT(48–60) [3, 4]. They bind to the lipid membrane with a high amount of anionic lipids. Membrane leakage is not observed at low micromolar concentrations. 

naCPPs and saCPPs are both less toxic than paCPPs, and higher concentrations or application of a transmembrane potential seems to be required to make the membrane unstable, both in the cell and in membrane model systems. It has been shown that acylation of these cationic peptides to make them more hydrophobic is a way to induce membrane leakage by this class of CPPs [20]. 

Table 1 shows examples of CPPs that have been studied and mentioned in this paper together with some of their physical properties.

## 3. Methods to Study CPP Uptake and the Mechanism(s) of Uptake

Despite many studies made on CPPs, the mechanism(s) by which CPPs enter the cells has not been completely resolved. Different biological and biophysical methods have been utilized to study the cellular uptake mechanism(s) and to follow CPPs and their conjugates inside the cells. There is no specific method which could give a complete answer for all questions. Therefore, a combination of different methods, model systems and techniques are required.

### 3.1. Biological Methods Using Cell Cultures

#### 3.1.1. Methods to Study Quantitative CPP and/or Cargo Uptake

The most common method to evaluate CPP uptake is by coupling a peptide to a fluorophore and measuring the fluorescence of treated cells. It is a convenient method and has been used to study both localization and amount of uptake of CPPs. Drawbacks to this method are that uptake does not always correlate with bioavailability, and care must therefore be taken when interpreting results of these kinds of studies. Furthermore, cationic CPPs are known to bind to the outside of the cell membrane and can thereby give false positive results, as fluorescence analysis cannot discriminate between internalized or surface bound CPPs. Protocols to reduce signals from surface bound CPPs include treating cells with trypsin to remove surface bound CPPs [21] and fluorescence quenching of surface bound fluorophores.

Fluorescence-assisted cell sorting (FACS) is a frequently used method to quantitatively measure uptake of labeled CPPs. A cell sorter apparatus sorts cells based upon the intensity of fluorescence and measures the amount of cells that have taken up the CPPs. However, FACS analysis cannot discriminate between surface bound and translocated fluorescence, so the above-mentioned protocols must be used. 

In live cells, by confocal microscopy imaging, one is able to monitor intracellular localization of CPPs or associated cargo molecules taken up by the cells. Using this method, it is possible to discriminate between extracellular and internalized peptides inside, for example, endosomes or the nucleus. Limitations associated with confocal microscopy are associated with statistical problems (due to small number of cells monitored), cost, and expertise needed to perform the experiments.

Functional assays can be performed to observe biological responses as well as evaluating their mechanism of uptake. One such method is the Cre-recombinase assay. It makes use of Cre-mediated recombination of the loxP-STOP-loxP EGFP-reporter gene giving rise to expression of EGFP. The recombination can only take place upon exogenous Cre-protein delivery to the nucleus [22]. The splice switching assay is another useful functional assay developed by Kole and coworkers [23, 24]. In this method, the cell lines are stably transfected with a plasmid carrying a luciferase gene and having an aberrant splice site. If a complementary ON could block this splice site, correct splicing of the luciferase gene towards the functional luciferase can be achieved. Luciferase activity measurements indicate the successful delivery of ON reaching the target. Internalization of the ON is achieved by using different CPPs.

#### 3.1.2. Methods to Study Molecular Mechanism of Uptake

There are also several other experimental methods to qualitatively investigate the cellular uptake mechanism. By inhibiting one or more pathways, it is in principle possible to find the mechanism(s) responsible for the uptake. One common way to assess involvement of endocytosis is treatment of cells with peptide at 4°C which inhibits all energy-dependent pathways [25].

Specific endocytosis inhibitors are frequently used to determine the mechanism of uptake of CPPs. Using such inhibitors, it was shown that uptake of TAT(48–60) was inhibited by cytochalasin D, an inhibitor of macropinocytosis [26]. These results were corroborated by another study, where uptake of TAT fusion protein was inhibited by amiloride, another inhibitor of macropinocytosis [22]. There are problems with using inhibitors to determine mechanism of uptake as the inhibitor might not be fully specific [27]. Also, shutting down one uptake route might induce uptake through another mechanism that is normally inactive. 

For investigation of uptake mechanisms, colocalization with endocytosis markers has also been studied. This method can also be used to determine the intracellular fate of CPPs. For example, lysotracker red, a substance that emits light in acidic conditions, can be used to determine if CPPs colocalize with lysosomes [28, 29].

Nowadays, several studies have used the effect of chloroquine (CQ) as an inhibitor of endosomal acidification and promoting the efficiency of the CPP. In addition, CQ is also applied to provide evidence for an endocytotic pathway. The lysosomotropic agent, CQ, is a relatively hydrophobic weak base with two basic groups. CQ operates by opposing the pH drop inside the endosome resulting in inhibition of endosome/lysosome fusion. Therefore, the macromolecule will be in the endosome for a longer period of time [30, 31]. When the result of a functional assay is affected by CQ, one may conclude that endocytosis is at least part of the CPP mechanisms. 

Pyrenebutyrate (PB) is an aromatic, hydrophobic, and negatively charged molecule that interacts electrostatically as a counterion with positively charged CPPs, particularly with the guanidinium group on arginine residues. The effect of PB on cellular uptake, translocation, as well as interaction of CPPs with model lipid membranes has been studied to investigate the mechanism of uptake. Upon interaction with hydrophilic oligoarginine peptides, the hydrophobicity is increased, and a direct membrane translocation is promoted according to the current mechanistic model [32–35]. When PB affects the results of a functional assay, one may conclude that membrane perturbations are rate limiting for the CPP activity, either in the endosomal escape process or in the direct penetration through the plasma membrane.

### 3.2. Biophysical Methods

Understanding the interactions of CPPs with model membranes or lipid bilayers contributes to the understanding of the mechanism(s) of the CPPs translocation [19]. For this purpose, different biophysical methods and model systems are available. Model membranes, particularly large unilamellar phospholipid vesicles (LUVs) are the most commonly used in the lipid-peptide interaction studies. Experimental conditions such as peptide concentration, membrane lipid composition, and charge of the lipids are important factors in biophysical studies of the uptake mechanism. 

The biophysical LUV leakage studies are indicators of the degree of perturbation to the membrane caused by different CPPs. The results are usually related to the direct penetration or endosomal escape of CPPs.

Some techniques such as circular dichroism, fluorescence and nuclear magnetic resonance are common biophysical spectroscopies that give more specific information about secondary structure, membrane interaction, and 3D-structure respectively. Molecular modeling is another method used for simulating the interaction of the CPP with the membrane.

## 4. Uptake Mechanisms

Despite some common properties of CPPs, especially their cationic nature, it is believed that the translocation mechanism is not the same for different families of CPPs. Also, most CPPs utilize two or more pathways depending on the experimental conditions. Here, we have briefly reviewed the two major cellular uptake mechanisms, nonendocytotic or energy-independent pathways and the endocytotic pathways.

### 4.1. Direct Penetration

Direct penetration via energy-independent pathways may include different mechanisms that have been described as inverted micelle formation [8], pore formation [36], the carpet-like model [37] and the membrane thinning model [38]. The first step in all these mechanisms constitutes interaction of the positively charged CPP with negatively charged components of membrane such as heparan sulfate (HS) as well as the phospholipid bilayer. They involve stable or transient destabilization of the membrane associated with folding of the peptide on the lipid membrane [12, 22, 39]. The subsequent mechanism of internalization depends highly on the peptide concentration, peptide sequence, and lipid composition in each model membrane study. 

Generally, direct penetration is most probable at high CPP concentrations and for primary amphiphatic CPPs such as transportan analogues and MPG [40–42].

The “inverted micelle” is one model suggested already at an early stage for the direct penetration of penetratin [43]. In addition to the interaction between the positively charged CPP and negatively charged components of the lipid membrane, interaction between hydrophobic residues such as tryptophan and the hydrophobic part of the membrane is also shown to be involved in this mechanism. Therefore, this mechanism is not probable for the highly cationic CPPs such as TAT(48–60).

Pore formation includes descriptions by the barrel stave model and the toroidal model [36]. In the barrel stave model, helical CPPs form a barrel by which hydrophobic residues are close to the lipid chains, and hydrophilic residues form the central pore. In the toroidal model, lipids bend in a way that the CPP is always close to the headgroup, and both CPP and lipids form a pore. In both mechanisms, pores appear when the concentration of the peptide is more than a certain concentration threshold, which is different for different peptides.

In the carpet-like model [37] and membrane thinning model [38], interactions between negatively charged phospholipid and cationic CPPs result in a carpeting and thinning of the membrane, respectively. Subsequent translocation of the CPP is achieved when CPP concentration is above a threshold concentration.

### 4.2. Endocytosis

**Figure 1:**
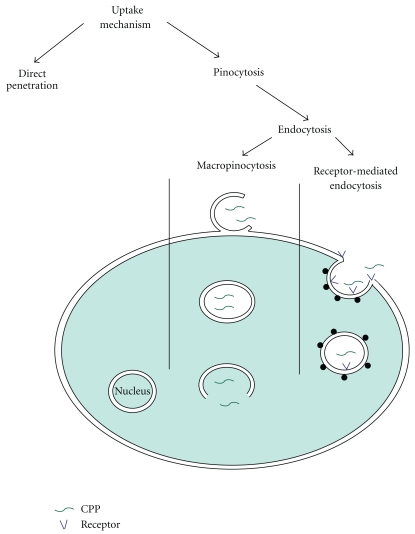
Scheme for different suggested uptake pathways for CPPs.

Endocytosis consists of several pathways including phagocytosis for uptake of large particles and pinocytosis for solute uptake. Pinocytosis is categorized as macropinocytosis, endocytosis dependent on the coat proteins clathrin or caveolin, or endocytosis independent of clathrin and/or caveolin (Figure 1) [44, 45]. 

Macropinocytosis is associated with the inward folding of the outer surface of the plasma membrane, which results in the formation of vesicles called macropinosomes. Resulting macropinosomes are surrounded by membrane similar to the cell membrane. Dynamin protein is required for membrane invagination. 

In receptor-mediated endocytosis, clathrin or caveolin pits are involved in the mechanism of uptake. Both clathrin and caveolin proteins cover the intracellular part of the membrane. They are required for invagination of the membrane and help to form the vesicles after binding the extracellular molecule to the membrane receptor. Clathrin-coated vesicles are about a few hundred nanometers in diameter, while caveolin-coated are about 50–80 nm in diameter [44, 45]. 

Earlier studies had suggested that direct penetration was the uptake mechanism for most CPPs. This conclusion was based on the observation that peptides enter the cell even at 4°C, therefore, by an energy-independent route. Later studies showed that experimental artifacts were responsible for this conclusion. Using methanol or formaldehyde to fix the investigated cells for confocal microscopy may result in some experimental artifacts [21, 46]. Nowadays by using trypsin to remove outside associated peptides and live cell confocal microscopy, one generally avoids this problem [21]. For most CPPs, it is now generally concluded that endocytosis is involved in the translocation mechanism. However it is most likely that different mechanisms operate under different conditions for all CPPs.

## 5. Factors Affecting the Mechanism of Cellular Uptake

In the study of the uptake mechanisms, both physicochemical properties of the CPP and the utilized experimental conditions are of importance.

Structure activity relationship (S.A.R) studies are able to recognize the importance of the individual residues in the CPP sequence. They have shown the importance of positive charges, especially arginine residues, in the uptake mechanism as well as hydrophobic alpha helical structures [11, 47, 48]. It has been shown that most CPPs are rich in arginine residues and that arginine (and in particular, its guanidinuim group) is more favorable than lysine for delivery and CPP activity of the peptides [12, 41, 49, 50]. However, this is not the case considering the high effect of TP10 and some other CPPs lacking arginine in their sequences. The CPP conformation and the length of the CPP sequence are other factors affecting the mechanism of uptake. This is shown by the difference between pVEC and scrambled pVEC in the uptake efficiency. The latter has no uptake, whereas the former efficiently translocates into various cell lines [48, 51]. Thermodynamic binding studies have shown that primary and secondary amphiphatic CPPs can directly penetrate through the cell membrane at low micromolar concentrations. However, non-amphiphatic CPPs mainly use endocytosis at low concentrations [17]. CPP conformations including induced alpha helices and beta sheets are also important in explaining the membrane perturbation and subsequent translocation by CPPs. 

Contradictory results are often reported which may arise from experimental conditions that differ in important respects. The first important factor is the CPP concentration, which affects the mechanism of CPP entry. Direct penetration is more probable for primary hydrophobic CPPs at high concentrations, whereas endocytosis is the main uptake mechanism at low concentrations. The concentration threshold for direct penetration varies between different CPPs, different cell lines, and the presence of and type of cargo.

It should be emphasized that the presence of the cargo may alter the CPP uptake pathway. Type of the cargo as well as the size and binding methodology have been shown to influence the CPP translocation mechanism. TAT attached to a large cargo is mostly entrapped in the endosomal vesicles; however, it redistributes throughout the cell cytosol when attached to a small cargo [52]. Furthermore, labeling a peptide with different fluorophores may also influence the uptake mechanism, intracellular distribution, and cytotoxicity of the peptide [53, 54]. Other experimental factors of importance for the uptake mechanisms are, for example, cell type, temperature, and incubation time.

## 6. Uptake Mechanism(s) for Selected CPPs

The CPPs described in Table 1 were divided into three different subgroups based on hydrophobicity, namely, hydrophilic or arginine-rich CPPs (R9 and TAT), intermediately hydrophobic CPPs (M918, pVEC, Penetratin and Pen-Arg) and a hydrophobic group, here with only one member (TP10). These peptides have different numbers of arginine residues, total positive charges, and hydrophobicity. They could use several different pathways for their cellular uptake and translocation. Generally, two major mechanisms have been mainly considered: the endosomal pathways composed of two steps, endocytotic entry followed by endosomal escape, and direct cell membrane penetration. The exact molecular pathways underlying their cellular uptake are not clear. Various studies have used different methods, inhibitors, tracer molecules, and model membranes to provide evidence for the priority of one or more pathways over the others. 

We have summarized important mechanistic results obtained from different reports on each investigated CPP.

### 6.1. Hydrophilic Arginine-Rich CPPs: R9 and TAT(48–60)

TAT(48–60) is derived from the transcription activating factor of human immunodeficiency virus (HIV-1) [4], while R9 is a synthetic homopolyarginine [11]. TAT(48–60), like R9, belongs to the hydrophilic group of CPPs but with a smaller number of arginine residues and more hydrophobicity. Arginine rich CPPs are able to deliver a variety of cargoes such as peptides, proteins, oligonucleotides, plasmid DNA and liposomes into mammalian cells in a functionally active form [55].

In the report by Guterstam et al. [56], direct penetration is suggested for the hydrophilic, arginine-rich R9, and TAT in the presence of high PB concentration (50 *μ*M). One reason is the observed diffuse intracellular distribution of the peptide as seen by confocal microscopy. However, at lower concentration of PB, as seen in confocal microscopy, the translocation of R9-bound ON mainly occurs by arginine-induced macropinocytosis, since CQ is required to obtain any splice-switching activity in the functional assay. In the splice-switching assay, the rate limiting step is endosomal escape which is promoted by hydrophobic counteranion PB. PB also increased to a small but significant extent the calcein leakage from POPC LUVs which indicates the probable contribution of pore formation or membrane perturbation for R9 translocation [56]. In several other studies, punctate cytoplasmic localization of labeled TAT and R9 observed by live cell imaging shows the participation of endocytosis in the uptake mechanism [21, 22, 57]. In contrast, another study has shown the presence of an energy- and temperature-independent pathway for some arginine-rich CPPs [12]. 

Similar observations are made in the presence of cargo. Recent studies have shown that different types of endocytotic pathways are involved in the uptake mechanism of arginine rich CPPs alone and when conjugated with the cargo molecules [22, 26, 55, 57–61]. The nature of the cell lines and the presence and type of the cargo may affect the specific mechanism employed by the CPP. 

Guterstam and coauthors describe that TAT-ON delivery is not as efficient as with R9 in the splice-switching assay. For TAT-ON, ON translocation through the endosome is probably a rate-limiting step in this assay. Similar to R9, the presence of PB has only little effect on calcein release from charged and uncharged LUVs [56].

Another important factor is the participation of heparan sulfate proteoglycans in the interaction between arginine rich CPPs and the cell membrane leading to cellular internalization [62–64].

### 6.2. Intermediately Hydrophobic CPPs; M918, Penetratin and pVEC

They all belong to secondary amphiphatic or intermediately hydrophobic CPPs (Table 1). pVEC is derived from the murine vascular endothelial-cadherin (VE-cadherin) protein. It has been shown that pVEC is able to translocate into different cell lines [13]. S.A.R studies have shown the effect of arginine and more importantly N-terminal hydrophobic residues in the translocation ability of pVEC both with and without the cargo [48]. Treatment of the cells with different endocytosis inhibitors efficiently suppresses the cellular uptake of pVEC. This effect is more pronounced for wortmannin indicating the presence of the clathrin dependent endocytotic pathway [48]. However uptake at low temperatures confirms the presence of non-endocytotic pathways in the pVEC uptake mechanism. The conjugate of pVEC with avidin translocates through the membrane by using clathrin dependent endocytosis, but the presence of another mechanism is also most likely under different conditions [57]. Guterstam et al. [56] describe the effect of PB on both biophysical and biological endpoints for pVEC. The fluorescent dye calcein leaked out significantly more from membrane vesicles (LUVs) in the presence than in the absence of PB, but the biological endpoints were not affected by PB. Therefore the rate limiting step for pVEC is probably related to the endocytotic entry rather than to the endocytotic escape. 

M918 is a novel CPP with 22 residues, seven of them positively charged. It is more hydrophilic than TP10 but more hydrophobic than penetratin (Table 1). M918 is able to deliver various cargo molecules into different cell lines. The presence of endocytosis inhibitors and lowering of temperature impaired the cellular internalization, confirming the presence of endocytotic pathways (especially macropinocytosis) in the uptake mechanism. However unlike for arginine rich CPPs, glycoaminoglycans on the cell membrane are not involved in cellular uptake mechanism. The same uptake mechanisms were observed in the absence or presence of cargo molecules. The splice correction assay confirms translocation and bioavailability of the cargo attached to the peptide [14]. 

Penetratin, the fragment of Antennapedia homeodomain with 16 residues is one of the most extensively used and studied CPPs [7]. It may be classified as an intermediately hydrophobic CPP [56]. However, in certain studies penetratin with seven positively charged residues has been classified as an arginine rich CPP. Therefore they share a common mechanism of cell entry with these CPPs. The majority of reports on the penetratin cellular uptake mechanism suggest that endocytosis is the major mechanism of uptake, both in the absence or presence of the cargo molecules. On the other hand, like arginine rich CPPs, different types of endocytotic pathways for penetratin and its cargo conjugates have been reported [22, 26, 55, 57–61]. Membrane perturbation studies using calcein leakage experiments revealed that penetratin also shares some properties with intermediately hydrophobic CPPs. Like pVEC and M918, penetratin caused calcein to leak out more quantitatively from membrane vesicles in the presence of PB, but PB had no effect in the splice correcting assay [56].

Pen-Arg is a penetratin analogue in which the lysines are exchanged to arginines. The cellular uptake of Pen-Arg and the splice switching activity of cargo conjugated to Pen-Arg was more efficient than the other intermediately hydrophobic CPPs in the presence of PB. This result indicates the importance of arginine residues in the interaction with cell membrane [56].

### 6.3. Hydrophobic CPP: TP10

TP10 is a transportan analogue in which first six N-terminal amino acids are removed. TP10 belongs to the primary hydrophobic CPPs with no arginine residue, and it shows less toxicity compared to transportan. Different cellular uptake and translocation mechanisms for TP10 and its cargo conjugates have been demonstrated. However, the translocation is suggested to proceed mainly via the endocytotic pathway [65]. Adding pyrenebutyrate had no effect on uptake efficiency and splice-switching activity of TP10, presumably due to the lack of arginine residues in its sequence [56]. In that study, hydrophobic TP10 acted completely in a different mode compared with other studied CPPs. It causes calcein leakage from membrane vesicles at very low peptide concentrations [66], and the leakage does not change in the presence of PB [56].

## 7. Native CPP-Like Peptides

Besides the native sequences that were originally found to be CPPs (from the TAT protein [3, 4] or the Antennapedia homeodomain [5–8]), there are other native peptide sequences that have been found to be CPPs. Three examples will be mentioned here.

The first category concerns the dynorphin neuropeptide family, where some members like big dynorphin or dynorphin A have been found to be very efficient CPPs [67]. They are relatively short, highly basic peptides which exert a number of important functions in the brain, mostly mediated by the kappa opioid receptor [68]. However, in addition the dynorphin neuropeptides are involved in so called nonopioid functions [69], which seem to depend on their ability to enter through cell membranes and find molecular interaction partners inside the cell. A speculative hypothesis makes this type of neuropeptides into the remains of an ancient signaling system, before the receptors were developed, when cell-to-cell signaling may have depended on the direct transfer of signaling molecules from one cell to the next.

A second example is taken from the prion protein, involved in infectious prion diseases affecting the brain, like the so-called “mad cow disease”. In diseased tissue, the protein is transformed from a normal cellular form into a so-called Scrapie form, which is aggregated and misfolded [70]. Peptide sequences from mouse or cow including the signal sequence (about 22 residues, mostly hydrophobic) followed by 6 N-terminal residues (mostly basic) of the prion protein have been characterized as CPPs [71]. In addition, the peptides seem to very specifically inhibit the formation of the Scrapie form of the protein inside the infected brain cells in a cell culture [72].

A third example is the human antibacterial peptide, LL-37. In addition to its antimicrobial activity which is associated to membrane damage, the peptide has been shown to act as a CPP in the eukaryotic host cells [73–75]. The different membrane activities have been explained by the different composition of the prokaryotic and eukaryotic membranes, with lower overall negative charge associated with the eukaryotic cell membranes.

## 8. CPPs and AMPs

AMPs (antimicrobial peptides) involved in host innate immunity share many structural aspects with CPPs. AMPs also consist of short sequences of cationic and hydrophobic peptides [76, 77]. Just like amphiphatic CPPs, upon binding to the target membrane (bacteria, viruses, fungi, and protozoa), they form amphiphatic structures. The presence of cationic residues in AMPs is vital, as it enhances electrostatic binding to the highly negatively charged membrane of their target, where the actual antimicrobial activity will begin. On the other hand, hydrophobic residues drive the entry of the peptide through the lipid bilayer. In contrast, the cell membrane of the CPP target is less anionic and has different compositions making it more resistant to AMPs [76]. 

The mechanism of action is not completely resolved for AMPs. Like CPPs, AMPs may use more than one mechanism of action depending on the nature of the AMP and the target. Mechanisms include transient or static pore formation and detergent-like solubilization. Different models have been proposed for describing these mechanisms: barrel-stave, toroid-pore, and the carpet-like models [76]. Recent work has shown the importance of the thermodynamics of the insertion of the peptide into the membrane in determination of the mechanism of action [78]. These models can also explain the transient membrane perturbation and subsequent cellular uptake for certain CPPs, especially primary amphiphatic CPPs such as transportan analogues [40–42]. There is a generally accepted view that association of the peptide to the membrane is the first step leading to destabilizing the membrane for both groups of peptides [12, 76]. The consequent cell entry highly depends on factors such as peptide and target properties. For certain CPPs which do not have high affinity for binding to or the ability to perturb the cell membrane, cellular uptake occurs via endocytosis as an alternative. In this case, the presence of proteogycans is necessary to promote the uptake [79]. The endocytotic pathway is not the entry route for AMPs as their bacterial targets lack factors necessary for the endocytotic pathway [76, 80]. As a result of these differences, AMPs have the ability to kill pathogenic agents, but most of them do not significantly harm the host cells. However, some AMPs are also toxic to the host cells [81]. In sharp contrast, CPPs are considered to internalize into the host cell and translocate different cargo molecules without causing any significant damage.

As a conclusion, these two groups of peptides are very similar from the molecular point of view, although they target different kinds of cell membranes. The observation that some CPPs such as TP10 and pVEC are also able to enter bacteria and act as antimicrobial peptides has made this group even more interesting [82]. On the other hand, some nonlytic AMPs have the ability to translocate across biological membrane of host cells in a nondisruptive way and even translocate cargo molecules into the cells [83]. Because of such similarities, some authors have put forward the idea that AMPs and CPPs could be classified as one group called membrane active peptides.

## 9. Conclusion

This paper concerns various aspects of CPPs, including their physicochemical properties, and mechanism(s) of cellular uptake and membrane translocation.

The CPPs represent a potentially valuable tool for the cellular delivery of important cargo molecules, considering their low toxicity and independence of membrane receptors and cell types. Since the discovery of the two well-known CPPs, the TAT and penetratin peptides, the number of known CPPs has considerably increased and their properties have been elucidated. Numerous preclinical applications for the treatment of certain diseases have been found due to the drug-delivery capabilities of the CPPs. 

Despite the similarities among CPPs, the mechanism(s) of their action may vary considerably. Contradictory observations indicate the presence of different factors which affect the cellular uptake and translocation mechanism(s). So far, most reports have pointed out endocytosis as the major cellular uptake pathway for most CPPs. However, there are still remaining important questions such as, what are the exact uptake mechanisms, how do different cargo molecules influence the cellular uptake of CPPs, and what are the additional aspects affecting the bioavailability of the CPPs? The answers to these questions may be found by systematic comparisons between uptake mechanisms of different CPPs both in the absence and presence of a variety of cargo molecules. 

## Figures and Tables

**Table 1:** Some CPPs and their physical properties. ^a^Hydrophobicity calculated according to the values from von Heijne scale [10].

Peptide	No. of arginines	No. of lysines	Hydrophobicity^a^	No. of residues	Sequence	Total charge
R9 [11]	9	—	2.58	9	RRRRRRRRR	+9
TAT(48–60) [4]	6	2	2.37	13	GRKKRRQRRRPPQ	+8
Penetratin [7]	3	4	1.52	16	RQIKIWFQNRRMKWKK	+7
Pen-Arg [12]	7	—	1.49	16	RQIRIWFQNRRMRWRR	+7
pVEC [13]	4	2	1.10	18	LLIILRRRIRKQAHAHSK	+8
M918 [14]	7	—	0.93	22	MVTVLFRRLRIRRACGPPRVRV	+7
TP10 [15]	—	4	0.53	21	AGYLLGKINLKALAALAKKIL	+4
